# Measles and Phenacetin: Which Killed the Patient—The Disease or the Treatment

**Published:** 1895-02

**Authors:** Charles E. Page

**Affiliations:** Boston, Mass.


### MEASLES AND PHENACETIN: WHICH KILLED THE PATIENT—THE DISEASE OR THE TREATMENT?

Charles E. Page, M. D., Boston, Mass.

In reporting a case of measles which proved fatal a writer in a current medical journal prefaces the history of the case with some thoughtful speculations concerning the possibly deleterious effects, at times and under some circumstances, of the coal-tar poisons.

"It would seem," he says, "that the marvelous potency of the pharmaceutical derivatives of coal-tar, at present in such general favor, should be sufficient to arouse the responsible physician to an unsual degree of alertness as to any deleterious effect upon the human organism which, when prescribed internally, they might naturally be expected capable of producing. Taking everything into consideration," he goes on to say, "it is quite remarkable that death in certain cases has not oftener been suspected of being the culmination of their use rather than, on the other hand, that they probably exerted the effect of at least postponing the occurrence of such a dire calamity."

From a purely speculative point of view he regarded it as incredible that these drugs, in doses ordinarily recommended, should be so much less liable to produce evil than good, "were it not for the results obtained from the extensive experience in their employment," in all of which the writer concurs, except in the view that the "results obtained" give us any rational hope that these poisons are ever "less liable to produce evil than good."

"Amid the complexities of a given instance of disease," further remarks Dr. Tyler, "it is practically impossible to clearly define that which may be designated the negative action of remedies employed. Observation and experience have too often avouched the efficiency of the *vis medicatrix naturæ*, for example, to avoid the acceptance of many apparent favorable results from artificial sources in any other spirit than that of extreme skepticism."

The case cited was that of an Italian, "about thirty-two years of age, muscular and exceptionally well-nourished." The historian remarked upon "the striking resemblance the symptoms bore in many respects to those which are quoted as having been observed in poisoning by Phenacetin," and he goes even further, in thinking that had the amounts employed been considerably greater the question might rationally have arisen "whether it was directly due to the complications resulting simply from the disease, to the remedy, or to a combination of the two," that death ensued. But the amount of Phenacetin was so small (fifty grains with whiskey in five days) that it seemed to him incredible that the drug could have much influenced the result.

But, he states that the second five-grain dose of Phenacetin on the fifteenth (the second day of sickness) reduced the fever markedly, from 103.6° to 101°, thus exhibiting a "marvelous potency" for either evil or good. Moreover, in spite of reduced temperature, "the pulse and respiration, on the other hand, became accelerated." My criticism here is, that a drug which increases the respiration and pulse of a patient, does so by adding to the toxic condition, giving the system still more to contend with, thus forcing it to work more vigorously because of the added load, not from increased strength, which being admitted tells, of course, against such a procedure. In this case, as usually happens—indeed, as it seems to me, must always be the case, in accordance with the laws of the animal organism, whatever the apparent effects at the moment, or the happy chance which allows of restoration from disease, under mistaken treatment the organism was made to work harder while the vital forces were actually depleted, the reduced temperature being, really, evidence of this; and the final and speedy exhaustion of the patient to his death in five days, though he was a muscular man, gives further and unmistakable evidence, as it seems to me, in the same direction

The patient was a splendid subject for water-treatment, and yet not one word is mentioned of even a cold compress over the region of the inflamed bronchi when, on the third day, "bronchitis became very evident in both lungs, and cough troublesomely aggravated." The constant changing of the cold compress over the chest in these cases (pneumonia, acute bronchitis, etc.) operates most charmingly. By this is not meant the application of a cold towel to remain for any length of time to become a hot fomentation, but its renewal every two or three minutes in desperate cases, and kept up till the conditions are markedly improved, and returned to at any time when evidence is observed of return of the congestion. Instead of this helpful and most regular (though not popular) treatment the chest was simply anointed with camphorated oil, an expectorant directed, and Quinin sulphate prescribed. Moreover, the patient was fed night and day, though he was at the start an exceptionally well-nourished patient and a promising subject for the fasting-cure; though, for that matter, it is my practice never to punish a fever patient with food, whether he be lean, stout or medium, or to offer any other nutriment than water, till convalescence is well established and satisfactory evidence observed—improved tongue, lowered temperature, good appetite, etc.—that the food stands a fair chance of being digested; otherwise it can serve no useful purpose, but must always prove a harmful nuisance, with absolutely no compensating effects. It tends to keep the patient's temperature up and going up, for it is so much more waste to be burned and eliminated. On the other hand, during every day of absolute fasting the toxic matters which are threatening life are reduced by about two pounds, and a few days of this sort of house-cleaning, as I have observed in innumerable instances, change the conditions so completely as to seem almost miraculous.

To quote: "At 4 A. M. of the 19th (sixth day of treatment), temperature 104°, Phenacetin and whiskey were again prescribed, but failed (as will be seen) of any salutary effect. At 5 A. M. the patient was noisily delirious and uneasy." What an indication for a Brand bath, to tranquillize the patient by means of the refreshing cold water, the effect of which is to strengthen the heart, brace the entire nervous system, prevent or restore from the dreaded coma! The common plan, however, of "silencing the alarm bells, instead of doing anything to put out the fire," was pursued: "Sol. morp. sulph. (U. S. P.), one fluid dram, which small quantity it was deemed advisable to supplement an hour later with Dover's powder, six grains. At 6.30 A. M., patient tranquil, but shortly lapsed into a comatose state from which it was only possible to partially arouse him. Pupils contracted; respiration varyingly noisy or stertorous; countenance cyanosed; extremities cold. Hypodermatic of Strychnin sulphate, one-thirtieth of a grain, and repeated two hours later (11 A. M.). Patient had become completely comatose and reflexly insensible to puncture of hypodermatic needle. He expired at 12.40 P. M. His temperature, taken one hour previously, was 106.8°."

The only water-treatment mentioned is that "at various times during the progress of the illness the surface was carefully sponged with alcohol and water combined." The general impression, both with the profession and laity, seems to be that a little water will not harm a fever-patient if it be well diluted with alcohol.

I have undertaken the study of this case in no spirit of carping, but to emphasize what seems the inevitably harmful routine procedures employed by the great majority of physicians in similar cases, as well as their lamentable failure in studying and applying the simple, but genuine, aids to nature supplied by the great variety of water-treatments.

From the study of many cases similar to the one here recorded, in treatment and results, as well as not a few which started out in similar fashion, but which under quite contrary methods resulted in speedy convalescence and recovery, it seems altogether probable that had this robust Italian been spared the ingestion of a single poisonous drug, as well as of all food-substances, which, from lack of possible digestion and assimilation, are themselves drugs, loading the circulation with the poisonous products of fermentation; and could he have had the benefit of appropriate baths—not for the purpose of downing the temperature, by any means, but, as has been already intimated, for their invigorating effects upon the entire organism—while the fluidity of the blood was maintained by plentiful supplies of pure soft water, hot and cold, the results would have been altogether satisfactory.—*Medical News*, November 24th, 1894.

